# 2453. Wastewater-Based Surveillance of Vancomycin-Resistant Enterococci in Hospitals

**DOI:** 10.1093/ofid/ofad500.2071

**Published:** 2023-11-27

**Authors:** Emily Au, Nicole Acosta, Barbara Waddell, Kristine Du, Maria Bautista, Janine McCalder, Jennifer Van Doorn, Kashtin Low, Rhonda Clark, Johann Pitout, Jenine Leal, Bayan Missaghi, Jamil Kanji, Oscar Larios, Elissa Rennert-May, Joseph Kim, Bonita Lee, Xiao-Li Pang, Kevin Frankowski, Casey R J Hubert, John Conly, Michael Parkins

**Affiliations:** University of Calgary, Calgary, Alberta, Canada; University of Calgary, Calgary, Alberta, Canada; University of Calgary, Calgary, Alberta, Canada; University of Calgary, Calgary, Alberta, Canada; University of Calgary, Calgary, Alberta, Canada; University of Calgary, Calgary, Alberta, Canada; University of Calgary, Calgary, Alberta, Canada; Mount Royal University, Calgary, Alberta, Canada; University of Calgary, Calgary, Alberta, Canada; University of Calgary, Calgary, Alberta, Canada; University of Calgary, Calgary, Alberta, Canada; University of Calgary, Calgary, Alberta, Canada; University of Alberta, Edmonton, Alberta, Canada; University of Calgary, Calgary, Alberta, Canada; University of Calgary, Calgary, Alberta, Canada; University of Calgary, Calgary, Alberta, Canada; University of Alberta, Edmonton, Alberta, Canada; University of Alberta, Edmonton, Alberta, Canada; University of Calgary, Calgary, Alberta, Canada; University of Calgary, Calgary, Alberta, Canada; University of Calgary, Calgary, Alberta, Canada; University of Calgary, Calgary, Alberta, Canada

## Abstract

**Background:**

Vancomycin-resistant *Enterococcus* (VRE) is an important nosocomial infection that may increase patient morbidity, mortality, and healthcare costs. We have adapted wastewater-based surveillance (WBS) as a novel tool to comprehensively and inclusively monitor the burden of VRE in tertiary acute care hospitals. Herein, we demonstrate our ability to detect, quantify and track VRE dynamically over time across a range of scales.

**Methods:**

Wastewater (WW) was collected from three hospitals in Calgary, AB: Rockyview General Hospital (RGH; 615 beds), Peter Lougheed Centre (PLC; 517 beds), and Foothills Medical Centre (FMC via three independent sites – A, B, C; 1100 beds). Three WW treatment plants (WWTPs; BBW, PCW, and FCW) serving the entire City of Calgary were sampled as community controls. DNA was extracted from WW pellets obtained following centrifugation. A multiplexed qPCR assay was adapted and used to quantify the abundances of the *vanA* and *vanB* resistance gene copies. Copy numbers were assessed as raw (copies per mL of WW processed) or normalized against three fecal biomarker genes: total bacterial 16S rRNA, human 18S rRNA, and *Bacteroides* HF183 16S rRNA. Differences between hospitals and controls were determined with Mann-Whitney tests (GraphPad Prism version 9.0).

**Results:**

Samples from the hospitals and WWTPs in Calgary, AB collected over 12 weeks demonstrated that all hospitals had 100-1000X higher mean aggregate abundances of both *vanA* and *vanB* relative to community-based WWTPs when assessed as raw or normalized by each fecal biomarker (Figure 1; only total bacterial 16S rRNA is shown, p< 0.001, Mann-Whitney). Within one individual hospital (RGH), each of *vanA* and *vanB* abundances follows similar trends over a 12-week period, regardless of whether the values were reported as raw or normalized with the three different fecal biomarker genes (Figure 2; only *vanA* is shown).

**Figure 1.**
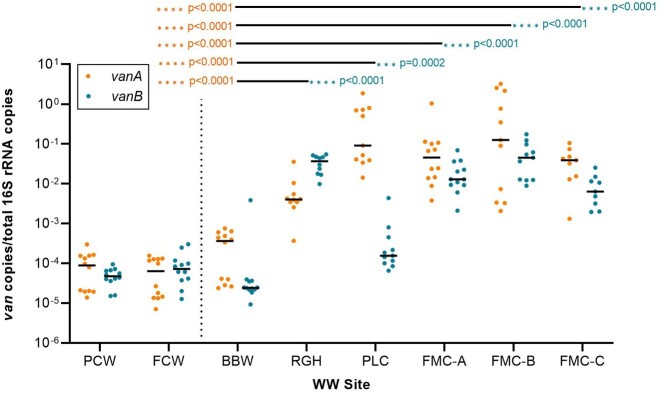
Mean aggregate copy numbers of vanA and vanB in wastewater measured by qPCR. VRE vanA and vanB gene abundances normalized as a ratio against total bacterial 16S rRNA copies demonstrate significantly higher abundances of both vanA and vanB in hospitals than the WWTP community controls.

**Figure 2.**
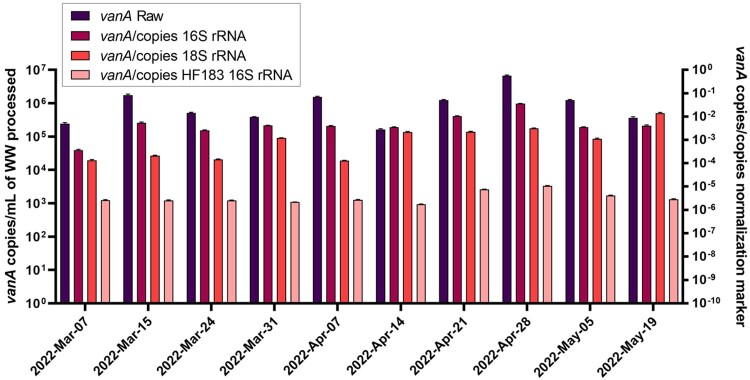
Comparison of vanA gene copies assessed as raw versus normalized as measured by qPCR from Rockyview General Hospital (RGH). VRE vanA gene abundances in Rockyview General Hospital (RGH) WW assessed as raw and normalized as a ratio against three different fecal biomarkers (total bacterial 16S rRNA, human 18S rRNA and Bacteroides HF183 16S rRNA) demonstrated similar trends in vanA gene abundances over time.

**Conclusion:**

WBS is a unique real-time tool that can be adapted to monitor the abundance of VRE across a range of scales. This tool has the potential to augment antimicrobial stewardship and infection prevention and control programs to better understand the contributing factors to selection and colonization.

**Disclosures:**

**All Authors**: No reported disclosures

